# Systematic reviews: knowledge translation?

**DOI:** 10.1590/2177-6709.21.1.013-014.edt

**Published:** 2016

**Authors:** Carlos Flores-Mir

**Affiliations:** * Professor and Orthodontic Graduate Program Director, University of Alberta

Translation is like a woman. 

If it is beautiful, it is not faithful. If it is faithful, it is most certainly not beautiful. Yevgeny Yevtushenko (Russian poet)

Systematic reviews are reproducible synthesis of the best available evidence to facilitate sounder clinical decisions. From the triad of factors that encompass evidence-based Dentistry - clinician's experience, patient's needs and preferences, available scientific evidence - systematic reviews clearly fit into the latter. What is important to underline is that scientific evidence alone should not direct patient care.

Why would any clinician prefer to consider a systematic review instead of any given individual paper? First and utmost because in only one manuscript the synthesis of "everything" published about that specific topic is available. That will avoid the clinician to have to look for, then pay for (in most cases) and finally, summarize, the information provided by innumerable papers. That sounds really enticing. There are some dangers involved in the process. One important one is to blindly accept any systematic review's conclusions. There are individuals like any of us involved in the process that can inadvertently make mistakes. Then there is a chance that not everything written about that specific topic is identified. Sometimes it is claimed that what is published is only the tip of an iceberg and that a lot of pertinent information is not easily retrievable and lays "under the visible water level." Finally, clinicians need to make a decision about how applicable is the provided information to the specific patients that they are facing their clinical chair.

The most important methodological characteristics that any systematic review should have are nicely summarized in the PRISMA guidelines.^1^It is strongly recommended to use them as closely as possible. They provide an easy to follow guide about how and where to report key characteristics of systematic reviews. In summary, the authors should provide a convincing argument that the topic they are presenting warrants a synthesis. If systematic reviews were already published in the area, a sound argument to why a new one is justifiable should be provided. The process should not only clearly explain where and how available evidence was identified, but also how it was selected. Then included papers should be individually criticized about any potential risk of bias through specific validated assessment tools. These processes should be completed at least in duplicate and independently. 

Meta-analysis should only be executed if available data warrant it, but most importantly because it makes clinical and methodological sense to combine the data. In addition, the risk of bias should be an integral part of the meta-analytic process. Sometimes reported meta-analysis results, from an unjustifiable synthesis process, could give readers a false sense of high quality of evidence. Nowadays the use of the GRADE recommendations starts to become a norm to help clinicians weight in the level of available evidence.^2^

From the clinician's point of view, synthesized information is great as it saves valuable time, but at the end of the day what is really important is how to translate the published conclusions consistently and fairly to our patients. That is an area that is not usually well crafted and reported in published systematic reviews. It is formally called "knowledge translation."

Uncertainty is the new name of the game. We have been focusing over the last century on mean amount of change. Nowadays we should consider an even more important piece of information - variability of individual response. In other words, what are the likely worst and best case scenarios. Here is where standard deviation and confidence intervals come into play. This should be an area that needs to be given more importance, as it allows clinicians to provide patients with realistic expectations, potential treatment outcomes. In this sense, well conducted meta-analyses allow us to report that data variability potential compared to simple qualitative reporting in systematic reviews without meta-analysis. 

Finally a philosophical question: Are systematic reviews more and more popular among researchers due to the right reason? Is it because it allows them to put relatively quickly a scientific publication without depending on factors related to the cost and time it will take to collect a study sample in a more typical study design? Or is it because there is a real quantifiable patient care impact in doing them? I strongly believe that it should be the second. Let's all together embrace this journey.

Later this year our journal DPJO will have an issue fully devoted to systematic reviews. We will be expecting your submissions. 

Carlos Flores-Mir - assistant editor (carlosflores@ualberta.ca)
